# A randomised, open-label, phase II study of tobemstomig combinations in patients with previously untreated advanced clear-cell renal cell carcinoma

**DOI:** 10.1016/j.esmoop.2026.108587

**Published:** 2026-10-09

**Authors:** B.I. Rini, T. Powles, P. Tomczak, S.J. Shin, J. Guo, J. Molina-Cerrillo, X. Ren, J.L. Lee, P.J. Wysocki, J. Puente, A. Basu, N. Mar, S. Bernhard, O. Khan, J. Ha, L. Colley, H. Prizant, L. Albiges

**Affiliations:** 1 Department of Medicine, Vanderbilt-Ingram Cancer Center, Nashville, USA; 2 Barts Cancer Centre, Barts Health NHS Trust, Queen Mary University of London, London, UK; 3 Department of Medical Oncology, Poznań University of Medical Sciences, Poznań, Poland; 4 Yonsei Cancer Center, Division of Medical Oncology, Department of Internal Medicine, Yonsei University College of Medicine, Seoul, Republic of Korea; 5 Department of Neurology and Melanoma, Peking University Cancer Hospital and Institute, Beijung, China; 6 Medical Oncology Department, Ramón y Cajal Health Research Institute (IRYCIS), CIBERONC, Alcalá University, University Hospital Ramon y Cajal, Madrid, Spain; 7 Department of Biotherapy, Tianjin Medical University Cancer Institute and Hospital, Tianjin, China; 8 Department of Oncology, Asan Medical Center, University of Ulsan College of Medicine, Seoul, Republic of Korea; 9 Department of Oncology, Jagiellonian University Medical College, Cracow, Poland; 10 Medical Oncology Department, Hospital Clínico San Carlos, Madrid, Spain; 11 Division of Hematology and Oncology, University of Alabama at Birmingham, Birmingham, USA; 12 Division of Hematology and Oncology, University of California at Irvine, Irvine, USA; 13 Roche Products Limited, Welwyn Garden City, UK; 14 Hoffmann-La Roche, Mississauga, Canada; 15 Genentech, Inc. South San Francisco, San Francisco, USA; 16 Department of Cancer Medicine, Institut Gustave Roussy, Villejuif, France

**Keywords:** immune checkpoint inhibitor, LAG3, renal cell carcinoma, TIGIT, tobemstomig, tyrosine kinase inhibitor

## Abstract

**Background:**

Immune checkpoint inhibitors combined with vascular endothelial growth factor (VEGF)-targeted tyrosine kinase inhibitors (TKIs) have improved advanced renal cell carcinoma (RCC) outcomes, yet median progression-free survival (PFS) remains <2 years.

**Patients and methods:**

This randomised (1 : 1 : 1), open-label, phase II study (NCT05805501) evaluated tobemstomig [bispecific anti–programmed cell death protein 1 (PD-1)/lymphocyte-activation gene 3] plus axitinib (VEGF-TKI) and tobemstomig plus tiragolumab (anti–T-cell immunoreceptor with immunoglobulin and immunoreceptor tyrosine-based inhibition motif domains) plus axitinib versus pembrolizumab (anti–PD-1) plus axitinib (control) in patients with untreated, locally advanced unresectable/metastatic clear-cell RCC with intermediate/poor International Metastatic Renal Cell Carcinoma Database Consortium risk. Following a protocol-specified interim futility analysis that led to study termination, the primary endpoint was amended from PFS and incidence/severity of adverse events (AEs) to the latter only. Efficacy was evaluated descriptively.

**Results:**

In the safety population (198 patients: tobemstomig-axitinib, *n* = 66; tobemstomig-tiragolumab-axitinib, *n* = 65; control, *n* = 67), grade 3-4 AEs occurred in 62.1%, 67.7% and 56.7% of patients in the respective arms and treatment-related serious AEs occurred in 22.7%, 29.2% and 13.4%. Confirmed overall response rates were 51.5%, 53.8% and 51.5%; median PFS was 12.4 [95% confidence interval (CI) 8.4-16.3], 12.5 (95% CI 7.3-17.2) and 12.5 months (95% CI 9.5 to not estimable).

**Conclusions:**

Tobemstomig-containing regimens were associated with increased toxicity and did not demonstrate meaningful efficacy improvement relative to control.

## Highlights

• Advanced RCC treated with first-line VEGF-TKI/anti-PD-1 combinations typically progresses within 2 years.
• Tobemstomig combinations showed no superior benefit to pembrolizumab-axitinib in intermediate/poor IMDC-risk RCC.
• There were more grade 3-4 AEs with tobemstomig combinations, particularly with tobemstomig-tiragolumab-axitinib.
• The findings did not support further development of tobemstomig-based regimens in advanced RCC.

## Introduction

The treatment landscape for advanced renal cell carcinoma (RCC) has evolved substantially with the introduction of immune checkpoint inhibitors (ICIs) and vascular endothelial growth factor (VEGF)-targeted tyrosine kinase inhibitors (TKIs), particularly in combination.^1^ For example, pembrolizumab [an anti–programmed cell death protein 1 (PD-1) antibody] plus axitinib (a VEGF receptor TKI) has demonstrated significantly improved overall survival (OS) and objective response rate (ORR) over sunitinib (a multitargeted TKI) in the first-line setting for advanced RCC.^2^

Despite these advances, median progression-free survival (PFS) with first-line ICI/TKI regimens is generally <2 years,^3^^,^^4^ underscoring the need for novel therapeutic strategies to enhance response durability and broaden clinical benefit.

Inhibition of novel immune checkpoints such as lymphocyte-activation gene 3 (LAG3) and T-cell immunoreceptor with immunoglobulin and immunoreceptor tyrosine-based inhibition motif domains (TIGIT) demonstrated promising early results in RCC.^5^^,^^6^ LAG3 and TIGIT contribute to T-cell exhaustion and resistance to PD-1 blockade.^7^^,^^8^ In RCC, high LAG3 and TIGIT expression correlates with immunosuppressive tumour microenvironments and poorer prognosis.^5^^,^^9^ The clinical relevance of these pathways is further supported by the U.S. Food and Drug Administration approval of nivolumab plus relatlimab (anti-LAG3) in advanced melanoma (based on the RELATIVITY-047 trial).^10^

This randomised phase II study (BO43936; NCT05805501) evaluated tobemstomig (bispecific anti-PD-1 and LAG3) and tiragolumab (an anti-TIGIT antibody), both combined with axitinib, versus the standard-of-care combination of pembrolizumab and axitinib in patients with previously untreated, advanced clear-cell RCC (ccRCC). Following a protocol-specified futility analysis, additional enrolment of patients into the study was discontinued. Here, we present safety and efficacy findings from the final descriptive analysis.

## Methods

This was a randomised, open-label, phase II study. The three treatment regimens were administered in repeated 21-day cycles until RECIST v1.1 disease progression, unacceptable toxicity or ≤24 months (whichever occurred first): tobemstomig 600 mg intravenously (i.v.) every 3 weeks (Q3W) plus axitinib 5 mg orally (p.o.) *biduum* two days in arm A, tobemstomig 600 mg i.v. Q3W plus tiragolumab 600 mg i.v. Q3W and axitinib 5 mg p.o. *biduum* two days in arm B and pembrolizumab 200 mg i.v. Q3W plus axitinib 5 mg p.o. *biduum* two days in the control arm.

Eligible patients were aged ≥18 years and had histologically confirmed RCC containing a clear-cell component (sarcomatoid features allowed), previously untreated, locally advanced, unresectable or metastatic disease measurable according to RECIST v1.1 and an International Metastatic Renal Cell Carcinoma Database Consortium (IMDC) risk score of intermediate or poor. Key exclusions were active central nervous system metastases, prior systemic therapy for advanced RCC or clinically meaningful autoimmune disease.

The study planned to enrol ∼210 participants, with primary endpoints of investigator-assessed PFS per RECIST v1.1 and incidence and severity of adverse events (AEs). A protocol-specified interim analysis for futility was conducted by an independent monitoring committee after the first 105 randomly allocated participants had ≥3 months of follow-up to stop one or both experimental arms if the ORR data indicated futility. The criterion to terminate an experimental arm was a prespecified difference in the confirmed ORR of >11% favouring the control arm. As a result of the futility findings, enrolment was terminated in all treatment arms after 199 patients had been randomly allocated. Subsequently, the sponsor discontinued the development of tobemstomig and the primary study endpoint was amended to incidence and severity of AEs only (protocol version 6). Ongoing treatment was permitted at the investigator’s discretion for patients deriving clinical benefit. Although the planned formal analysis of efficacy endpoints could not be carried out, a descriptive analysis of efficacy [ORR, PFS, duration of response (DOR) and OS] was completed in the efficacy-assessable population.

The trial was conducted according to Good Clinical Practice guidelines of the International Conference on Harmonisation and the principles of the Declaration of Helsinki. The trial protocol was approved by independent review boards or ethics committees at each of the study sites. All patients provided written informed consent.

## Results

The protocol-specified futility analysis (clinical cut-off date: 22 April 2024) was conducted in the first 105 enrolled patients with ≥3 months of follow-up. The tobemstomig plus axitinib arm crossed the futility boundary for ORR, with an observed difference of 11.44% favouring the control arm. As no meaningful efficacy advantage was seen in either of the experimental arm and numerically higher incidences of serious AEs (SAEs), grade 3-4 AEs and immune-mediated AEs were reported, further study enrolment was paused for all three arms.

### Patients

Between 21 April 2023 and 11 July 2024, 199 patients were enrolled and randomly allocated. At the data cut-off date for the final descriptive analysis (1 February 2025), the median follow-up was ∼11 months across the treatment arms (Table 1). The safety-assessable population included 198 patients (one randomly allocated patient did not receive any study treatment): 67 patients in the control arm, 66 patients in arm A and 65 patients in arm B. The efficacy-assessable population included 197 patients as two patients were mistakenly randomly allocated but did not meet the eligibility criteria (66, 66 and 65 patients, respectively; Supplementary Figure S1, available at https://doi.org/10.1016/j.esmoop.2026.108587). Approximately half of these patients remained on treatment at data cut-off; the most common reasons for treatment discontinuation were progressive disease (PD) in the control arm and arm A and AEs and PD in arm B.

**Table 1:** Efficacy in the efficacy-assessable population

Outcome	Arm A: tobemstomig + axitinib (*n* = 66)	Arm B: tobemstomig + tiragolumab + axitinib (*n* = 65)	Control: pembrolizumab + axitinib (*n* = 66)
Follow-up (months), median (range)	11.3 (1-20)	11.1 (2-21)	11.7 (1-21)
Confirmed ORR, *n* (%)	34 (51.5)	35 (53.8)	34 (51.5)
Complete response	0	2 (3.1)	0
Partial response	34 (51.5)	33 (50.8)	34 (51.5)
Stable disease	23 (34.8)	24 (36.9)	27 (40.9)
Progressive disease	4 (6.1)	4 (6.2)	3 (4.5)
NE	1 (1.5)	1 (1.5)	0
Missing	4 (6.1)	1 (1.5)	2 (3.0)
Unconfirmed ORR, *n* (%)	36 (54.5)	41 (63.1)	40 (60.6)
Responders, *n*	34	35	34
Responders with subsequent event, *n* (%)	11 (32.4)	12 (34.3)	10 (29.4)
Death, *n*	0	1	2
Disease progression, *n*	11	11	8
Duration of response (months), median (95% CI)	12.4 (8.5-NE)	14.7 (9.5-NE)	NE (9.6-NE)
PFS (months), median (95% CI)	12.4 (8.4-16.3)	12.5 (7.3-17.2)	12.5 (9.5-NE)

No missing data were imputed. Patients without postbaseline tumour assessments were considered nonresponders for ORR. Statistical analyses were carried out using SAS 9.4.

CI, confidence interval; NE, not estimable; ORR, objective response rate; PFS, progression-free survival.

Baseline demographics and disease characteristics were generally balanced (Table 2). Most patients had IMDC intermediate risk (81.7%; 161 of 197), stage IV disease at diagnosis (52.8%; 104 of 197), metastatic disease at enrolment (88.3%; 174 of 197) and ≥1% PD-L1 expression (63.6%; 110 of 173 of assessable patients).

**Table 2:** Baseline demographics and disease characteristics in the efficacy-assessable population

Characteristic	Arm A: tobemstomig + axitinib (*n* = 66)	Arm B: tobemstomig + tiragolumab + axitinib (*n* = 65)	Control: pembrolizumab + axitinib (*n* = 66)	All patients (*N* = 197)
Age (years), median (range)	61.0 (40-86)	62.0 (41-78)	61.5 (34-82)	61.0 (34-86)
Age, *n* (%)				
<65 years	38 (57.6)	38 (58.5)	40 (60.6)	116 (58.9)
≥65 years	28 (42.4)	27 (41.5)	26 (39.4)	81 (41.1)
Sex, *n* (%)				
Male	51 (77.3)	52 (80.0)	55 (83.3)	158 (80.2)
Female	15 (22.7)	13 (20.0)	11 (16.7)	39 (19.8)
Race, *n* (%)				
Asian	29 (43.9)	25 (38.5)	23 (34.8)	77 (39.1)
Black/African American	0	0	1 (1.5)	1 (0.5)
White	27 (40.9)	34 (52.3)	35 (53)	96 (48.7)
Unknown	1 (1.5)	0	1 (1.5)	2 (1.0)
Not reported	9 (13.6)	6 (9.2)	6 (9.1)	21 (10.7)
ECOG performance status, *n* (%)				
0	26 (39.4)	29 (44.6)	32 (48.5)	87 (44.2)
1	40 (60.6)	36 (55.4)	34 (51.5)	110 (55.8)
Karnofsky performance status score, *n*	66	63	65	194
70-80, *n* (%)	30 (45.5)	31 (49.2)	18 (27.7)	79 (40.7)
90-100, *n* (%)	36 (54.5)	32 (50.8)	47 (72.3)	115 (59.3)
IMDC risk (IxRS), *n* (%)				
Intermediate	54 (81.8)	53 (81.5)	54 (81.8)	161 (81.7)
Poor	12 (18.2)	12 (18.5)	12 (18.2)	36 (18.3)
Smoking status, *n* (%)				
Current/former	25 (37.9)	30 (46.2)	34 (51.5)	89 (45.2)
Never	41 (62.1)	35 (53.8)	32 (48.5)	108 (54.8)
Initial diagnosis staging, *n* (%)				
Stage I	6 (9.1)	12 (18.5)	8 (12.1)	26 (13.2)
Stage II	13 (19.7)	5 (7.7)	4 (6.1)	22 (11.2)
Stage III	13 (19.7)	14 (21.5)	18 (27.3)	45 (22.8)
Stage IV	34 (51.5)	34 (52.3)	36 (54.5)	104 (52.8)
Disease status at enrolment, *n* (%)				
Metastatic	57 (86.4)	60 (92.3)	57 (86.4)	174 (88.3)
Locally advanced	9 (13.6)	5 (7.7)	9 (13.6)	23 (11.7)
Prior cancer radiotherapy, *n*	66	65	66	197
Yes, *n* (%)	8 (12.1)	5 (7.7)	10 (15.2)	23 (11.7)
No, *n* (%)	58 (87.9)	60 (92.3)	56 (84.8)	174 (88.3)
Prior nephrectomy, *n* (%)				
Yes	31 (47.0)	33 (50.8)	29 (43.9)	93 (47.2)
No	35 (53.0)	32 (49.2)	37 (56.1)	104 (52.8)
PD-L1 status by SP263 (TAP score), *n*	57	57	59	173
Negative (<1%), *n* (%)	15 (26.3)	23 (40.4)	25 (42.4)	63 (36.4)
Positive (≥1%), *n* (%)	22 (38.6)	21 (36.8)	21 (35.6)	64 (37.0)

ECOG, Eastern Cooperative Oncology Group; IMDC, International Metastatic Renal Cell Carcinoma Database Consortium; IxRS, interactive voice and web response system; PD-L1, programmed death-ligand 1; SP263, Ventana PD-L1 (SP263) assay (Ventana Medical Systems, Tucson, AZ); TAP, tumour area positivity.

### Safety overview

Treatment exposure is summarised in Supplementary Table S1 (available at https://doi.org/10.1016/j.esmoop.2026.108587). Grade 3-4 AEs occurred in 56.7% (38 of 67) of patients in the control arm, 62.1% (41 of 66) of patients in arm A, and 67.7% (44 of 65) of patients in arm B (Table 3). Grade 5 AEs were reported in 3.5% (7 of 198) of patients overall (Table 3). AEs were sepsis (control arm); atrioventricular block, gastrointestinal obstruction and haemoptysis (arm A) and stroke, unexplained death and suicide (arm B); none were considered treatment-related. SAEs were reported in 31.3% (21 of 67), 42.4% (28 of 66) and 61.5% (40 of 65) of patients in the control arm, arm A and arm B, respectively (Table 3). Treatment-related SAEs were observed in 13.4% (control 9 of 67), 22.7% (arm A 15 of 66) and 29.2% (arm B 19 of 65) of patients (Table 3; Supplementary Table S2, available at https://doi.org/10.1016/j.esmoop.2026.108587). Treatment-related AEs leading to discontinuation of any study treatment were observed in 11.9% (control 8 of 67), 12.1% (arm A 8 of 66) and 29.2% (arm B 19 of 65) of patients (Supplementary Table S3, available at https://doi.org/10.1016/j.esmoop.2026.108587).

**Table 3:** Safety summary in the safety-assessable population

AE	Arm A: tobemstomig + axitinib (*n* = 66)	Arm B: tobemstomig + tiragolumab + axitinib (*n* = 65)	Control: pembrolizumab + axitinib (*n* = 67)	All patients (*N* = 198)
Total number of patients (%) with ≥1 AE	65 (98.5)	64 (98.5)	66 (98.5)	195 (98.5)
Total number of AEs, *n*	777	784	784	2345
Grade 3-4 AE, *n* (%)	41 (62.1)	44 (67.7)	38 (56.7)	123 (62.1)
SAE, *n* (%)	28 (42.4)	40 (61.5)	21 (31.3)	89 (44.9)
Treatment-related SAE, *n* (%)	15 (22.7)	19 (29.2)	9 (13.4)	43 (21.7)
Grade 5 AE, *n* (%)^a^	3 (4.5)^b^	3 (4.6)^c^	1 (1.5)^d^	7 (3.5)
Immune-mediated AE, *n* (%)	50 (75.8)	52 (80.0)	47 (70.1)	149 (75.3)
AE leading to withdrawal from any study treatment, *n* (%)	8 (12.1)	19 (29.2)	10 (14.9)	37 (18.7)
AE leading to withdrawal of:				
Pembrolizumab, *n* (%)	0	0	6 (9.0)	6 (3.0)
Tiragolumab, *n* (%)	0	17 (26.2)	0	17 (8.6)
Tobemstomig, *n* (%)	8 (12.1)	18 (27.7)	0	26 (13.1)
Axitinib, *n* (%)	2 (3.0)	6 (9.2)	7 (10.4)	15 (7.6)

AEs were graded using the National Cancer Institute Common Terminology Criteria for Adverse Events v5.0 in the safety-assessable population (all randomised patients who received at least one dose of any study treatment).

AE, adverse event; SAE, serious adverse event.

a None of these grade 5 AEs were related to the study treatment.

b Atrioventricular block, gastrointestinal obstruction and haemoptysis.

c Stroke, unexplained death and suicide.

d Sepsis.

Overall, higher incidences of grade 3-4 AEs, SAEs, immune-mediated AEs and AEs leading to treatment withdrawal were observed in arm B, followed by arm A and the control arm (Table 3). The most common clinically relevant grade 3-5 AEs, treatment-related SAEs and immune-mediated AEs are summarised in the Supplementary Results (available at https://doi.org/10.1016/j.esmoop.2026.108587).

### Efficacy

Confirmed ORRs were observed in 51.5% of patients (34 of 66) in the control arm, 51.5% of patients (34 of 66) in arm A and 53.8% of patients (35 of 65) in arm B (Table 1). Notably, arm B included two patients with complete responses (CRs), whereas all other responses across arms were partial responses. The median DOR was not estimable [NE; 95% confidence interval (CI) 9.6-NE] in the control arm, 12.4 months (95% CI 8.5-NE) in arm A and 14.7 months (95% CI 9.45-NE) in arm B (Figure 1B).

**Figure 1:**
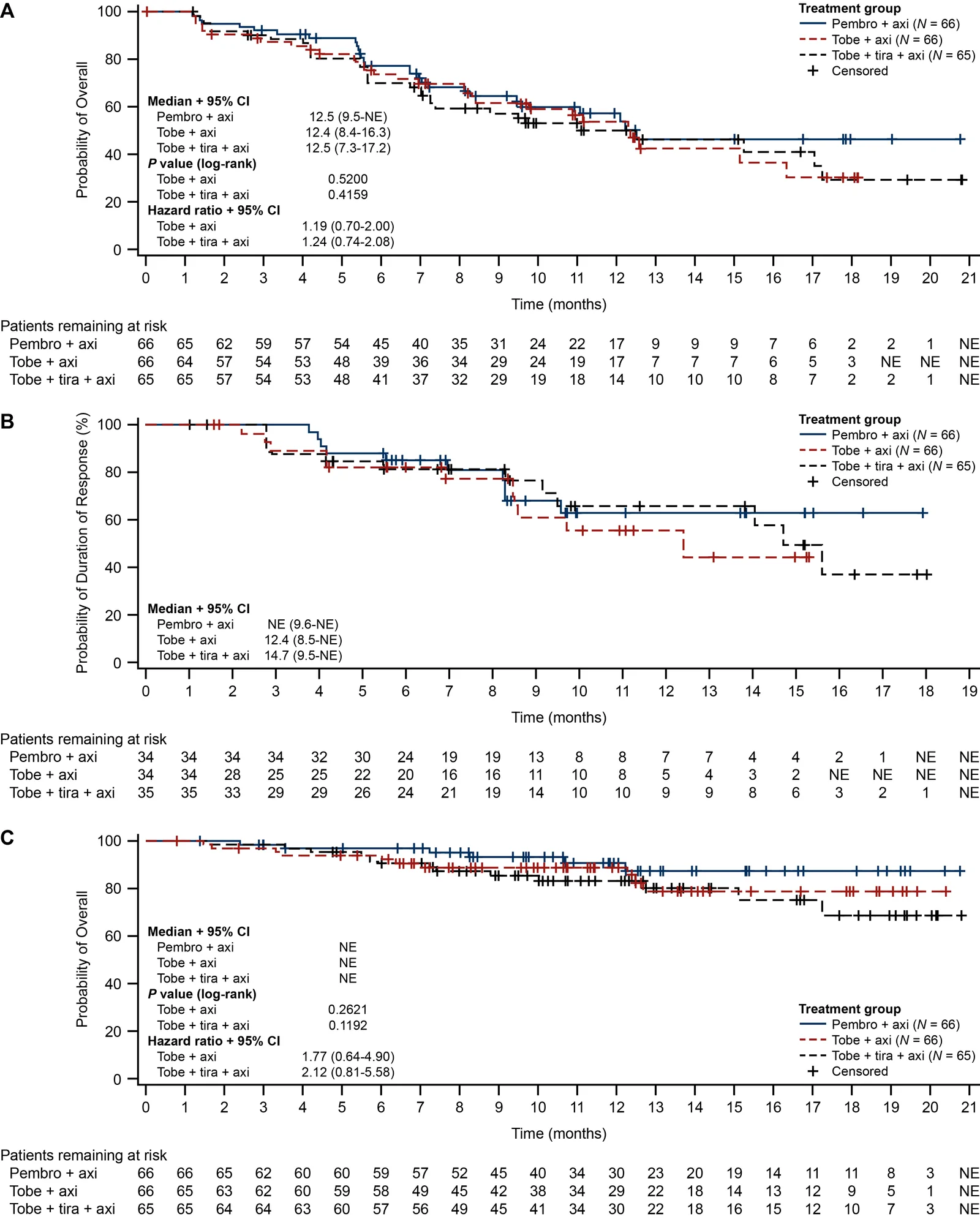
**(A) PFS, (B) DOR and (C) OS (efficacy-assessable population).** No missing data were imputed. Patients without postbaseline tumour assessments were considered nonresponders for ORR. Statistical analyses were carried out using SAS 9.4. axi, axitinib; CI, confidence interval; DOR, duration of response; NE, not estimable; ORR, objective response rate; OS, overall survival; pembro, pembrolizumab; PFS, progression-free survival; tira, tiragolumab; tobe, tobemstomig.

Median PFS was 12.5 months (95% CI 9.5-NE) in the control arm, 12.4 months (95% CI 8.4-16.3) in arm A and 12.5 months (95% CI 7.3-17.2) in arm B (Table 1, Figure 1A).

Median OS was not reached in any arm; the hazard ratio versus the control arm was 1.8 (95% CI 0.6-4.9) for arm A and 2.1 (95% CI 0.8-5.6) for arm B (Figure 1C). Six-month landmark event-free survival rates were 96.9% (95% CI 92.6-100.0) in the control arm, 93.8% (95% CI 87.9-99.7) in arm A and 90.6% (95% CI 83.5-97.8) in arm B, reflecting slightly lower rates in the experimental arms than in the control arm. Additional analyses of ORR and PFS stratified by IMDC risk factors are presented in Supplementary Figure S2 (available at https://doi.org/10.1016/j.esmoop.2026.108587).

## Discussion

Novel approaches are needed to build upon the benefits of immune-based combinations in metastatic RCC. Approaches targeting LAG3 and TIGIT represent viable candidates in RCC given the underlying immune-responsive biology and effect observed in other solid tumours. In this randomised phase II study in patients with untreated, advanced ccRCC, although the overall incidence of AEs was similar among all three arms, the tobemstomig-containing regimens led to numerically higher incidences of clinically relevant AEs. The triplet regimen was associated with higher rates of grade 3-4 AEs, SAEs and AE-related treatment discontinuations compared with the doublet. The grade 5 AEs in arm B were unrelated to treatment and did not prompt additional safety monitoring or protocol amendments before enrolment was halted. These findings highlight the challenges of intensifying established PD-1/VEGF-based regimens with additional immune checkpoint agents, in which increased toxicity may limit the potential for clinical benefit in unselected patient populations. The tobemstomig combinations did not demonstrate a clinical benefit over control, with no meaningful PFS or ORR improvements. Tobemstomig, as a monotherapy and combination therapy, has been assessed in other phase Ib/II studies, including non-small-cell lung cancer (NCT05775289)^11^ and melanoma (NCT05116202),^12^ with no improved clinical outcomes compared with ICI-based regimens. Therefore, the development of tobemstomig was discontinued.

The response rates observed in this study were broadly consistent with previously reported outcomes for anti-PD-1–VEGF-TKI combinations.^2^^,^^13^ Although the interim futility analysis showed a numerically lower ORR for tobemstomig plus axitinib compared with control, the final analysis showed similar confirmed ORRs (51.5% versus 51.5%). This apparent difference likely reflects the impact of a smaller patient sample size and a shorter follow-up period used in the futility analysis. Nevertheless, the final analysis did not demonstrate a meaningful efficacy advantage for either of the tobemstomig-containing regimen. The observed ORRs were similar across arms [51.5% (arm A and the control) and 53.8% (arm B)] and the low CR rates across the experimental arms indicate that tobemstomig with or without tiragolumab did not confer greater antitumour activity compared with established anti-PD-1–VEGF-TKI combinations such as pembrolizumab plus axitinib. The relatively modest activity, particularly the low CR rate and similar progression patterns across arms, may reflect the inherent limitations of PD-1 pathway inhibition, whether alone or in combination with additional checkpoint inhibitors, in this treatment setting.

Our findings echo broader challenges seen in achieving added benefit through triplet regimens in renal cancer. In STELLAR-002, the triplet combination of zanzalintinib (selective VEGF receptor TKI) plus nivolumab (anti-PD-1) and relatlimab (LAG-3 inhibitor) demonstrated a lower ORR (33%) compared with the zanzalintinib plus nivolumab doublet (63%) in the first-line treatment of ccRCC, with similar disease control rates and higher discontinuation due to toxicity, suggesting no added benefit from the triplet strategy in unselected patients.^14^ In COSMIC-313, cabozantinib plus nivolumab and ipilimumab improved PFS compared with nivolumab plus ipilimumab but did not demonstrate an OS benefit and was associated with increased toxicity and reduced treatment exposure.^15^ These findings reinforce the notion that triplet regimens, particularly those incorporating TIGIT- or LAG-3-directed agents, may not yield incremental clinical benefit over established ICI and VEGF-targeted combinations in unselected patients.

The observed response data and primary disease progression rates, although broadly consistent with prior studies,^2^^,^^15^^,^^16^ may reflect differences in patient selection, particularly with respect to risk stratification and baseline tumour characteristics. All patients in the current study were classified as IMDC intermediate or poor risk, unlike many phase III trials that enrolled patients across all IMDC risk groups, including favourable-risk populations known to have better outcomes and longer survival. These nuances highlight the need for deeper biomarker-driven understanding of response and resistance mechanisms in ccRCC. High levels of CD8+ T-cell infiltration in ccRCC are associated with an unfavourable prognosis,^17^^,^^18^ in contrast with melanoma, suggesting that these T cells are profoundly dysfunctional or ‘fixed’ in an exhausted state, and not readily reversible by single or dual checkpoint blockade. Deciphering this state will require spatial multi-omic profiling to isolate the specific pathways driving this resistance. Future strategies should prioritise novel mechanisms of action beyond current PD-1/VEGF backbones, including the addition of hypoxia-inducible factor-2 alpha inhibitors, metabolic or myeloid modulators, antibody–drug conjugates and engineered T-cell platforms.^19^^,^^20^

In conclusion, the addition of tobemstomig to axitinib demonstrated no added benefit compared with standard of care in patients with previously untreated, advanced ccRCC. The triplet combination of tobemstomig, tiragolumab and axitinib was associated with increased toxicity without meaningful improvement in efficacy. These findings, alongside results from other triplet studies and the plateauing benefit of current PD-1/VEGF-based regimens, highlight the need to reconsider development strategies and prioritise innovative, biology-guided approaches in renal cancer.

## Supplementary data

Supplementary Table S3

Supplementary Results
