# Supplementary material for: A randomised, open-label, phase II study of tobemstomig combinations in patients with previously untreated advanced clear-cell renal cell carcinoma
Source: ESMO Open. 2026 Oct 9;11(10):108587. doi: 10.1016/j.esmoop.2026.108587 (PMC13675702; doi:10.1016/j.esmoop.2026.108587)
Supplement: Supplementary file 1 [file mmc1.docx]

**Supplementary Figure 1. Trial profile**

^a^ Patients were randomised 1:1:1 using an interactive voice or web-based response system (IxRS) using a permuted-block method. The allocation sequence was generated and managed automatically by IxRS, with no sponsor or investigator access. Randomisation was stratified by International Metastatic Renal Cell Carcinoma Database Consortium (IMDC) risk (intermediate versus poor) [Heng DY et al, J Clin Oncol, 2009].
