# Supplementary material for: A randomised, open-label, phase II study of tobemstomig combinations in patients with previously untreated advanced clear-cell renal cell carcinoma
Source: ESMO Open. 2026 Oct 9;11(10):108587. doi: 10.1016/j.esmoop.2026.108587 (PMC13675702; doi:10.1016/j.esmoop.2026.108587)
Supplement: Supplementary file 2 [file mmc2.docx]

**Supplementary Figure 2. (A) Best confirmed ORR in Arm A and the control arm, (B) PFS in Arm A and the control arm by IMDC risk, (C) best confirmed ORR in Arm B and the control arm, (D) PFS in Arm B and the control arm (efficacy-evaluable population)**

Axi, axitinib; IMDC, International Metastatic Renal Cell Carcinoma Database Consortium; NE, not evaluable; ORR, objective response rate; PFS, progression-free survival; pembro, pembrolizumab; Tira, tiragolumab; Tobe, tobemstomig.
