# Supplementary material for: A randomised, open-label, phase II study of tobemstomig combinations in patients with previously untreated advanced clear-cell renal cell carcinoma
Source: ESMO Open. 2026 Oct 9;11(10):108587. doi: 10.1016/j.esmoop.2026.108587 (PMC13675702; doi:10.1016/j.esmoop.2026.108587)
Supplement: Supplementary file 3 [file mmc3.docx]

**Supplementary Table 1.** **Treatment exposure by arm in the safety-evaluable population**

|  | **Arm A:**  **Tobemstomig +** **axitinib (*n* = 66)** | **Arm B:**  **Tobemstomig + tiragolumab + axitinib (*n* = 65)** | | **Control:**  **Pembrolizumab + axitinib (*n* = 67)** |
| --- | --- | --- | --- | --- |
|  | **Axitinib** | **Axitinib** | | **Axitinib** |
| **Treatment duration, days** | | | | |
| Mean (SD) | 276.1 (164.7) | 258.6 (160.2) | | 297.8 (166.5) |
| Median | 254.0 | 249.0 | | 283.0 |
| Min–Max | 1–621 | 1–632 | | 20–631 |
| **Dose intensity, %** | | | | |
| Mean (SD) | 86.85 (18.71) | 90.10 (16.27) | | 88.64 (17.66) |
| Median | 94.3 | 96.08 | | 96.00 |
| Min–Max | 29.2–133.9 | 52.3–150.0 | | 23.0–119.6 |
| **Number of doses received** | | | | |
| Mean (SD) | 481.7 (308.2) | 444.3 (278.9) | | 516.0 (322.6) |
| Median | 465.0 | 393.0 | | 488.0 |
| Min–Max | 2–1176 | 2–1180 | | 40–1226 |
| **Total cumulative dose, mg** | | | | |
| Mean (SD) | 2156.85 (1375.30) | 1990.86 (1213.65) | | 2442.82 (1704.32) |
| Median | 1953.00 | 1981.00 | | 2225.00 |
| Min–Max | 10.0–6006.0 | 10.0–5900.0 | | 200.0–8005.0 |
|  | **Tobemstomig** | **Tobemstomig** | **Tiragolumab** | **Pembrolizumab** |
| **Treatment duration, days** | | | | |
| Mean (SD) | 235.0 (170.0) | 218.1 (169.5) | 219.7 (168.6) | 276.3 (169.5) |
| Median | 236.0 | 176.0 | 176.0 | 253.0 |
| Min–Max | 1–620 | 1–632 | 1–632 | 1–631 |
| **Dose intensity, %** | | | | |
| Mean (SD) | 95.70 (8.32) | 90.97 (11.71) | 90.41 (12.98) | 93.70 (10.74) |
| Median | 99.19 | 96.18 | 96.18 | 98.95 |
| Min–Max | 66.7–104.0 | 54.0–102.4 | 44.9–102.4 | 45.7–101.5 |
| **Number of doses received** | | | | |
| Mean (SD) | 11.5 (7.6) | 10.2 (7.4) | 10.2 (7.4) | 13.2 (8.0) |
| Median | 12.0 | 8.0 | 8.0 | 12.0 |
| Min–Max | 1–28 | 1–31 | 1–31 | 1–31 |
| **Total cumulative dose, mg** | | | | |
| Mean (SD) | 6872.73  (4530.73) | 6104.31 (4418.34) | 6103.62 (4416.58) | 2644.78  (1590.43) |
| Median | 7200.00 | 4800.00 | 4800.00 | 2400.00 |
| Min–Max | 600.0–  16800.0 | 600.0–18600.0 | 600.0– 18600.0 | 200.0–  6200.0 |

Max, maximum; min, minimum; SD, standard deviation.

Treatment duration is the date of the last dose of study medication minus the date of the

first dose plus 1 day. Dose intensity is the number of doses actually received divided by the

number of expected doses.
