# Supplementary material for: A randomised, open-label, phase II study of tobemstomig combinations in patients with previously untreated advanced clear-cell renal cell carcinoma
Source: ESMO Open. 2026 Oct 9;11(10):108587. doi: 10.1016/j.esmoop.2026.108587 (PMC13675702; doi:10.1016/j.esmoop.2026.108587)
Supplement: Supplementary file 4 [file mmc4.docx]

**Supplementary Table 2.** **SAEs related to any study treatment in the safety evaluable population**

|  | **Arm A: Tobemstomig + axitinib**  **(*n* = 66)** | **Arm B: Tobemstomig + tiragolumab + axitinib (*n* = 65)** | **Control: Pembrolizumab + axitinib (*n* = 67)** | **All patients (*n* = 198)** |
| --- | --- | --- | --- | --- |
| Patients with at least one AE, *n* (%) | 15 (22.7) | 19 (29.2) | 9 (13.4) | 43 (21.7) |
| Number of events, *n* | 22 | 30 | 12 | 64 |
| Gastrointestinal disorders |  |  |  |  |
| Patients with at least one AE, *n* (%) | 3 (4.5) | 3 (4.6) | 2 (3.0) | 8 (4.0) |
| Number of events, *n* | 3 | 4 | 3 | 10 |
| Colitis, *n* (%) | 1 (1.5) | 1 (1.5) | 0 | 2 (1.0) |
| Diarrhoea, *n* (%) | 1 (1.5) | 0 | 1 (1.5) | 2 (1.0) |
| Vomiting, *n* (%) | 0 | 1 (1.5) | 1 (1.5) | 2 (1.0) |
| Abdominal pain, *n* (%) | 0 | 1 (1.5) | 0 | 1 (0.5) |
| Enterocolitis, *n* (%) | 0 | 0 | 1 (1.5) | 1 (0.5) |
| Functional gastrointestinal disorder, *n* (%) | 1 (1.5) | 0 | 0 | 1 (0.5) |
| Nausea, *n* (%) | 0 | 1 (1.5) | 0 | 1 (0.5) |
| Investigations |  |  |  |  |
| Patients with at least one AE, *n* (%) | 4 (6.1) | 3 (4.6) | 1 (1.5) | 8 (4.0) |
| Number of events, *n* | 5 | 4 | 1 | 10 |
| Alanine aminotransferase increased, *n* (%) | 2 (3.0) | 2 (3.1) | 1 (1.5) | 5 (2.5) |
| Aspartate aminotransferase increased, *n* (%) | 2 (3.0) | 1 (1.5) | 0 | 3 (1.5) |
| Transaminases increased, *n* (%) | 1 (1.5) | 1 (1.5) | 0 | 2 (1.0) |
| Hepatobiliary disorders |  |  |  |  |
| Patients with at least one AE, *n* (%) | 4 (6.1) | 2 (3.1) | 1 (1.5) | 7 (3.5) |
| Number of events, *n* | 4 | 2 | 1 | 7 |
| Hepatitis, *n* (%) | 3 (4.5) | 0 | 0 | 3 (1.5) |
| Hypertransaminasaemia, *n* (%) | 0 | 2 (3.1) | 1 (1.5) | 3 (1.5) |
| Drug-induced liver injury, *n* (%) | 1 (1.5) | 0 | 0 | 1 (0.5) |
| Cardiac disorders |  |  |  |  |
| Patients with at least one AE, *n* (%) | 2 (3.0) | 3 (4.6) | 1 (1.5) | 6 (3.0) |
| Number of events, *n* | 2 | 4 | 1 | 7 |
| Myocarditis, *n* (%) | 1 (1.5) | 2 (3.1) | 1 (1.5) | 4 (2.0) |
| Autoimmune myocarditis, *n* (%) | 0 | 1 (1.5) | 0 | 1 (0.5) |
| Cardiac failure, *n* (%) | 1 (1.5) | 0 | 0 | 1 (0.5) |
| Myocardial infarction, *n* (%) | 0 | 1 (1.5) | 0 | 1 (0.5) |
| General disorders and administration-site conditions |  |  |  |  |
| Patients with at least one AE, *n* (%) | 2 (3.0) | 3 (4.6) | 0 | 5 (2.5) |
| Number of events, *n* | 2 | 4 | 0 | 6 |
| Fatigue, *n* (%) | 1 (1.5) | 1 (1.5) | 0 | 2 (1.0) |
| Mucosal inflammation, *n* (%) | 1 (1.5) | 1 (1.5) | 0 | 2 (1.0) |
| Asthenia, *n* (%) | 0 | 1 (1.5) | 0 | 1 (0.5) |
| Endocrine disorders |  |  |  |  |
| Patients with at least one AE, *n* (%) | 0 | 2 (3.1) | 2 (3.0) | 4 (2.0) |
| Number of events, *n* | 0 | 2 | 2 | 4 |
| Adrenal insufficiency, *n* (%) | 0 | 1 (1.5) | 1 (1.5) | 2 (1.0) |
| Glucocorticoid deficiency, *n* (%) | 0 | 0 | 1 (1.5) | 1 (0.5) |
| Primary adrenal insufficiency, *n* (%) | 0 | 1 (1.5) | 0 | 1 (0.5) |
| Metabolism and nutrition disorders |  |  |  |  |
| Patients with at least one AE, *n* (%) | 2 (3.0) | 1 (1.5) | 1 (1.5) | 4 (2.0) |
| Number of events, *n* | 2 | 1 | 1 | 4 |
| Acidosis, *n* (%) | 0 | 0 | 1 (1.5) | 1 (0.5) |
| Decreased appetite, *n* (%) | 1 (1.5) | 0 | 0 | 1 (0.5) |
| Hyponatraemia, *n* (%) | 0 | 1 (1.5) | 0 | 1 (0.5) |
| Type 1 diabetes mellitus, *n* (%) | 1 (1.5) | 0 | 0 | 1 (0.5) |
| Injury, poisoning and procedural complications |  |  |  |  |
| Patients with at least one AE, *n* (%) | 2 (3.0) | 1 (1.5) | 0 | 3 (1.5) |
| Number of events, *n* | 2 | 1 | 0 | 3 |
| Infusion-related reaction, *n* (%) | 1 (1.5) | 1 (1.5) | 0 | 2 (1.0) |
| Medication error, *n* (%) | 1 (1.5) | 0 | 0 | 1 (0.5) |
| Nervous system disorders |  |  |  |  |
| Patients with at least one AE, *n* (%) | 1 (1.5) | 2 (3.1) | 0 | 3 (1.5) |
| Number of events, *n* | 1 | 2 | 0 | 3 |
| Cerebral infarction, *n* (%) | 1 (1.5) | 0 | 0 | 1 (0.5) |
| Cerebrovascular accident, *n* (%) | 0 | 1 (1.5) | 0 | 1 (0.5) |
| Immune-mediated encephalitis, *n* (%) | 0 | 1 (1.5) | 0 | 1 (0.5) |
| Blood and lymphatic system disorders |  |  |  |  |
| Patients with at least one AE, *n* (%) | 0 | 1 (1.5) | 1 (1.5) | 2 (1.0) |
| Number of events, *n* | 0 | 1 | 1 | 2 |
| Anaemia, *n* (%) | 0 | 0 | 1 (1.5) | 1 (0.5) |
| Immune thrombocytopenia, *n* (%) | 0 | 1 (1.5) | 0 | 1 (0.5) |
| Renal and urinary disorders |  |  |  |  |
| Patients with at least one AE, *n* (%) | 1 (1.5) | 1 (1.5) | 0 | 2 (1.0) |
| Number of events, *n* | 1 | 1 | 0 | 2 |
| Acute kidney injury, *n* (%) | 1 (1.5) | 0 | 0 | 1 (0.5) |
| Prerenal failure, *n* (%) | 0 | 1 (1.5) | 0 | 1 (0.5) |
| Respiratory, thoracic and mediastinal disorders |  |  |  |  |
| Patients with at least one AE, *n* (%) | 0 | 1 (1.5) | 1 (1.5) | 2 (1.0) |
| Number of events, *n* | 0 | 1 | 1 | 2 |
| Epistaxis, *n* (%) | 0 | 1 (1.5) | 0 | 1 (0.5) |
| Immune-mediated lung disease, *n* (%) | 0 | 0 | 1 (1.5) | 1 (0.5) |
| Immune system disorders |  |  |  |  |
| Patients with at least one AE, *n* (%) | 0 | 1 (1.5) | 0 | 1 (0.5) |
| Number of events, *n* | 0 | 1 | 0 | 1 |
| Anaphylactic shock, *n* (%) | 0 | 1 (1.5) | 0 | 1 (0.5) |
| Infections and infestations |  |  |  |  |
| Patients with at least one AE, *n* (%) | 0 | 1 (1.5) | 0 | 1 (0.5) |
| Number of events, *n* | 0 | 1 | 0 | 1 |
| Cholecystitis infective, *n* (%) | 0 | 1 (1.5) | 0 | 1 (0.5) |
| Skin and subcutaneous tissue disorders |  |  |  |  |
| Patients with at least one AE, *n* (%) | 0 | 0 | 1 (1.5) | 1 (0.5) |
| Number of events, *n* | 0 | 0 | 1 | 1 |
| Pruritus, *n* (%) | 0 | 0 | 1 (1.5) | 1 (0.5) |
| Vascular disorders |  |  |  |  |
| Patients with at least one AE, *n* (%) | 0 | 1 (1.5) | 0 | 1 (0.5) |
| Number of events, *n* | 0 | 1 | 0 | 1 |
| Hypertension, *n* (%) | 0 | 1 (1.5) | 0 | 1 (0.5) |

AE, adverse event; SAE, serious adverse event.
