# Supplementary material for: A randomised, open-label, phase II study of tobemstomig combinations in patients with previously untreated advanced clear-cell renal cell carcinoma
Source: ESMO Open. 2026 Oct 9;11(10):108587. doi: 10.1016/j.esmoop.2026.108587 (PMC13675702; doi:10.1016/j.esmoop.2026.108587)
Supplement: Supplementary file 5 [file mmc5.docx]

**Supplementary Table 3.** **Treatment-related AEs leading to discontinuation of any study treatment by treatment arm in the safety evaluable population**

|  | **Arm A: Tobemstomig + axitinib**  **(*n* = 66)** | **Arm B: Tobemstomig + tiragolumab + axitinib (*n* = 65)** | **Control: Pembrolizumab + axitinib (*n* = 67)** | **All patients (*n* = 198)** |
| --- | --- | --- | --- | --- |
| Patients with at least one AE, n (%) | 8 (12.1) | 19 (29.2) | 8 (11.9) | 35 (17.7) |
| Number of events, *n* | 8 | 24 | 10 | 42 |
| Hepatobiliary disorders |  |  |  |  |
| Patients with at least one AE, *n* (%) | 3 (4.5) | 3 (4.6) | 2 (3.0) | 8 (4.0) |
| Number of events, *n* | 3 | 3 | 3 | 9 |
| Hepatitis, *n* (%) | 3 (4.5) | 0 | 1 (1.5) | 4 (2.0) |
| Hypertransaminasaemia, *n* (%) | 0 | 2 (3.1) | 1 (1.5) | 3 (1.5) |
| Drug-induced liver injury, *n* (%) | 0 | 1 (1.5) | 0 | 1 (0.5) |
| Cardiac disorders |  |  |  |  |
| Patients with at least one AE, *n* (%) | 1 (1.5) | 4 (6.2) | 0 | 5 (2.5) |
| Number of events, *n* | 1 | 4 | 0 | 5 |
| Myocarditis, *n* (%) | 1 (1.5) | 2 (3.1) | 0 | 3 (1.5) |
| Autoimmune myocarditis, *n* (%) | 0 | 1 (1.5) | 0 | 1 (0.5) |
| Myocardial infarction, *n* (%) | 0 | 1 (1.5) | 0 | 1 (0.5) |
| Gastrointestinal disorders |  |  |  |  |
| Patients with at least one AE, *n* (%) | 2 (3.0) | 3 (4.6) | 0 | 5 (2.5) |
| Number of events, *n* | 2 | 3 | 0 | 5 |
| Colitis, *n* (%) | 1 (1.5) | 2 (3.1) | 0 | 3 (1.5) |
| Diarrhoea, *n* (%) | 1 (1.5) | 1 (1.5) | 0 | 2 (1.0) |
| Investigations |  |  |  |  |
| Patients with at least one AE, *n* (%) | 2 (3.0) | 2 (3.1) | 1 (1.5) | 5 (2.5) |
| Number of events, *n* | 2 | 5 | 2 | 9 |
| Alanine aminotransferase increased, *n* (%) | 0 | 1 (1.5) | 1 (1.5) | 2 (1.0) |
| Aspartate aminotransferase increased, *n* (%) | 0 | 1 (1.5) | 1 (1.5) | 2 (1.0) |
| Transaminases increased, *n* (%) | 1 (1.5) | 1 (1.5) | 0 | 2 (1.0) |
| Blood bilirubin increased, *n* (%) | 0 | 1 (1.5) | 0 | 1 (0.5) |
| Hepatic enzyme increased, *n* (%) | 1 (1.5) | 0 | 0 | 1 (0.5) |
| Skin and subcutaneous tissue disorders |  |  |  |  |
| Patients with at least one AE, *n* (%) | 0 | 1 (1.5) | 3 (4.5) | 4 (2.0) |
| Number of events, *n* | 0 | 1 | 3 | 4 |
| Pruritus, *n* (%) | 0 | 1 (1.5) | 1 (1.5) | 2 (1.0) |
| Psoriasis, *n* (%) | 0 | 0 | 1 (1.5) | 1 (0.5) |
| Rash, *n* (%) | 0 | 0 | 1 (1.5) | 1 (0.5) |
| Blood and lymphatic system disorders |  |  |  |  |
| Patients with at least one adverse event, *n* (%) | 0 | 1 (1.5) | 0 | 1 (0.5) |
| Number of events, *n* | 0 | 1 | 0 | 1 |
| Immune thrombocytopenia, *n* (%) | 0 | 1 (1.5) | 0 | 1 (0.5) |
| Endocrine disorders |  |  |  |  |
| Patients with at least one AE, *n* (%) | 0 | 1 (1.5) | 0 | 1 (0.5) |
| Number of events, *n* | 0 | 1 | 0 | 1 |
| Primary adrenal insufficiency, *n* (%) | 0 | 1 (1.5) | 0 | 1 (0.5) |
| Eye disorders |  |  |  |  |
| Patients with at least one AE, *n* (%) | 0 | 0 | 1 (1.5) | 1 (0.5) |
| Number of events, *n* | 0 | 0 | 1 | 1 |
| Eye haemorrhage, *n* (%) | 0 | 0 | 1 (1.5) | 1 (0.5) |
| General disorders and administration-site conditions |  |  |  |  |
| Patients with at least one AE, *n* (%) | 0 | 1 (1.5) | 0 | 1 (0.5) |
| Number of events, *n* | 0 | 1 | 0 | 1 |
| Mucosal inflammation, *n* (%) | 0 | 1 (1.5) | 0 | 1 (0.5) |
| Immune system disorders |  |  |  |  |
| Patients with at least one AE, *n* (%) | 0 | 1 (1.5) | 0 | 1 (0.5) |
| Number of events, *n* | 0 | 1 | 0 | 1 |
| Anaphylactic shock, *n* (%) | 0 | 1 (1.5) | 0 | 1 (0.5) |
| Infections and infestations |  |  |  |  |
| Patients with at least one AE, *n* (%) | 0 | 1 (1.5) | 0 | 1 (0.5) |
| Number of events, *n* | 0 | 1 | 0 | 1 |
| Sinusitis, *n* (%) | 0 | 1 (1.5) | 0 | 1 (0.5) |
| Injury, poisoning and procedural complications |  |  |  |  |
| Patients with at least one AE, *n* (%) | 0 | 1 (1.5) | 0 | 1 (0.5) |
| Number of events, *n* | 0 | 1 | 0 | 1 |
| Infusion-related reaction, *n* (%) | 0 | 1 (1.5) | 0 | 1 (0.5) |
| Musculoskeletal and connective tissue disorders |  |  |  |  |
| Patients with at least one AE, *n* (%) | 0 | 0 | 1 (1.5) | 1 (0.5) |
| Number of events, *n* | 0 | 0 | 1 | 1 |
| Arthralgia, *n* (%) | 0 | 0 | 1 (1.5) | 1 (0.5) |
| Nervous system disorders |  |  |  |  |
| Patients with at least one AE, *n* (%) | 0 | 1 (1.5) | 0 | 1 (0.5) |
| Number of events, *n* | 0 | 1 | 0 | 1 |
| Immune-mediated encephalitis, *n* (%) | 0 | 1 (1.5) | 0 | 1 (0.5) |
| Renal and urinary disorders |  |  |  |  |
| Patients with at least one AE, *n* (%) | 0 | 1 (1.5) | 0 | 1 (0.5) |
| Number of events, *n* | 0 | 1 | 0 | 1 |
| Chronic kidney disease, *n* (%) | 0 | 1 (1.5) | 0 | 1 (0.5) |

AE, adverse event.
