# Supplementary material for: A randomised, open-label, phase II study of tobemstomig combinations in patients with previously untreated advanced clear-cell renal cell carcinoma
Source: ESMO Open. 2026 Oct 9;11(10):108587. doi: 10.1016/j.esmoop.2026.108587 (PMC13675702; doi:10.1016/j.esmoop.2026.108587)
Supplement: Supplementary file 6 [file mmc6.docx]

**Supplementary Results**

***Safety Summary***

Across treatment arms, the only grade 3–4 AEs that consistently met the ≥3 patient threshold in all arms were alanine aminotransferase (ALT) increases, hypertension, and palmar-plantar erythrodysaesthesia syndrome. ALT increases were observed in 3 patients (4.5%) in the tobemstomig + axitinib arm, 6 patients (9.2%) in the tobemstomig + tiragolumab + axitinib arm, and 5 patients (7.5%) in the pembrolizumab + axitinib arm. Hypertension was reported in 6 patients (9.1%) in the tobemstomig + axitinib arm, 5 patients (7.7%) in the tobemstomig + tiragolumab + axitinib arm, and 7 patients (10.4%) in the pembrolizumab + axitinib arm. Palmar-plantar erythrodysaesthesia syndrome was observed in 3 patients (4.5%) in tobemstomig + axitinib arm, 4 patients (6.2%) in the tobemstomig + tiragolumab + axitinib arm, and 3 patients (4.5%) in the pembrolizumab + axitinib arm.

No treatment-related SAE met the ≥3 patient threshold across all arms for any preferred term. However, treatment-related SAEs of hepatitis occurred in 3 patients (4.5%) in the tobemstomig + axitinib arm.

Immune-mediated AEs that met the ≥10 patient threshold in all arms were hepatic, dermatologic, and endocrine toxicities.

- Immune-mediated hepatitis (diagnosis and lab abnormalities) was reported in 28 patients (42.4%) in the tobemstomig + axitinib arm, 28 patients (43.1%) in the tobemstomig + tiragolumab + axitinib arm, and 26 patients (38.8%) in the pembrolizumab + axitinib arm. Within this category, ALT increases occurred in 17 (25.8%), 18 (27.7%), and 16 (23.9%) patients, and AST increases in 15 (22.7%), 20 (30.8%), and 12 (17.9%) patients, respectively.
- Immune-mediated hepatitis (lab abnormalities) was reported in 27 patients (40.9%) in the tobemstomig + axitinib arm, 27 patients (41.5%) in the tobemstomig + tiragolumab + axitinib arm, and 24 patients (35.8%) in the pembrolizumab + axitinib arm. Within this category, ALT increases occurred in 17 (25.8%), 18 (27.7%), and 16 (23.9%) patients, and AST increases in 15 (22.7%), 20 (30.8%), and 12 (17.9%) patients, respectively.
- Immune-mediated rash was observed in 27 patients (40.9%) in the tobemstomig + axitinib arm, 29 patients (44.6%) in the tobemstomig + tiragolumab + axitinib arm, and 22 patients (32.8%) in the pembrolizumab + axitinib arm. Palmar-plantar erythrodysaesthesia syndrome was reported in 18 (27.3%), 10 (15.4%), and 15 (22.4%) patients, and rash was reported in 12 (18.2%), 17 (26.2%), and 10 (14.9%) patients, respectively.
- Immune-mediated hypothyroidism was reported in 25 patients (37.9%) in the tobemstomig + axitinib arm, 25 patients (38.5%) in the tobemstomig + tiragolumab + axitinib arm, and 22 patients (32.8%) in the pembrolizumab + axitinib arm. Within this category, hyperthyroidism was reported in 18 (27.3%), 20 (30.8%) and 17 (25.4%) patients, respectively.
